# 2289: Will the Veteran Affairs (VA) electronic medical records (EMR) database reveal a signal that angiotensin II inhibiting medications ameliorate depression?

**DOI:** 10.1017/cts.2017.66

**Published:** 2018-05-10

**Authors:** David D. Maron, Marc Blackman, Richard Amdur, Thomas Mellman, Kathryn Sandberg

## Abstract

OBJECTIVES/SPECIFIC AIMS: Angiotensin type 1 receptor blockers (ARBs) and angiotensin-converting enzyme inhibitors (ACEIs) are frequently prescribed for hypertension and associated cardiovascular and renal complications. In animal models, these drugs also reduce anxiety and depression. OBJECTIVE—to determine if Veteran Affairs (VA) clinical pharmacy data indicate a protective effect of ARBs and/or ACEIs for major depression. METHODS/STUDY POPULATION: Pharmacy records from nationwide VA electronic medical records (EMR) were extracted for patients prescribed ARBs, ACEIs, α-blockers, β-blockers, calcium channel blockers, or diuretics (n=4,081,359). Patients were excluded if: they had not received medications for 6 months with >70% coverage; were diagnosed with substance/alcohol abuse, dementia, psychosis, schizophrenia, or prescribed insulin. The study population was categorized as “ARB/ACEI” (A/A) or “Never ARB/ACEI” (NA/A). Using the Greedy Matching Algorithm, subjects were matched on a 1:1 ratio for sex and race over a 5 year age range resulting in 2 equal groups of n=1,350,236 each. Subjects were older (M=71.6, SD=12) and mostly men (97%). RESULTS/ANTICIPATED RESULTS: In the A/A Versus NA/A, respectively, the incidence of anti-depressant use was greater during (9.9% vs. 8.9%) but was lower after (11.8% vs. 12.2%) the study period. PHQ-2 scores (Mean±SD) were statistically lower, albeit similar, during (0.79±1.56 vs. 0.85±1.63) and after (1.00±1.73 vs. 1.07±1.79) the study period. DISCUSSION/SIGNIFICANCE OF IMPACT: These preliminary data suggest that inhibiting angiotensin II action does not provide a protective effect on major depression when compared with other classes of antihypertensive drugs. This study illustrates how “Big Data” may inform the design, or obviate the need, for large-scale randomized-controlled trials.

